# 2-Amino-4,6-dimethyl­pyrimidinium chloro­acetate

**DOI:** 10.1107/S1600536809044444

**Published:** 2009-10-31

**Authors:** Cui-Hua Lin, Nai-Sheng Liu, Fang-Fang Jian

**Affiliations:** a Microscale Science Institute, Department of Chemistry and Chemical Engineering, Weifang University, Weifang 261061, People’s Republic of China; b Journal Editorial Department, Weifang University, Weifang 261061, People’s Republic of China; c Microscale Science Institute, Weifang University, Weifang 261061, People’s Republic of China

## Abstract

There are two cations and two anions in the asymmetric unit of the title compound, C_6_H_10_N_3_^+^·C_2_H_2_ClO_2_^−^. In the crystal, the components are linked by inter­molecular N—H⋯O and N—H⋯N hydrogen bonds to form a two-dimensional network. Additional stabilization is provided by weak inter­molecular C—H⋯O inter­actions.

## Related literature

For background to pyrimidine derivatives, see: Xue *et al.* (1993 ▶); Hemamalini *et al.* (2005 ▶). 
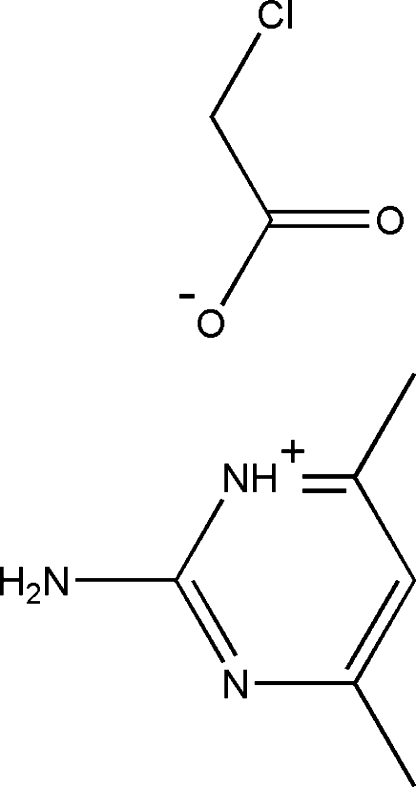


## Experimental

### 

#### Crystal data

C_6_H_10_N_3_^+^·C_2_H_2_ClO_2_^−^
*M* *_r_* = 217.66
Triclinic, 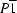

*a* = 4.4560 (9) Å
*b* = 12.302 (3) Å
*c* = 19.441 (4) Å
α = 92.90 (3)°
β = 96.53 (3)°
γ = 91.15 (3)°
*V* = 1057.1 (4) Å^3^
*Z* = 4
Mo *K*α radiation
μ = 0.34 mm^−1^
*T* = 293 K
0.20 × 0.15 × 0.11 mm

#### Data collection

Bruker SMART CCD diffractometer
Absorption correction: none
10303 measured reflections
4761 independent reflections
3452 reflections with *I* > 2σ(*I*)
*R*_int_ = 0.028

#### Refinement

*R*[*F* ^2^ > 2σ(*F* ^2^)] = 0.055
*wR*(*F* ^2^) = 0.195
*S* = 1.08
4761 reflections
253 parameters
H-atom parameters constrained
Δρ_max_ = 0.42 e Å^−3^
Δρ_min_ = −0.43 e Å^−3^

### 

Data collection: *SMART* (Bruker, 1997 ▶); cell refinement: *SAINT* (Bruker, 1997 ▶); data reduction: *SAINT*; program(s) used to solve structure: *SHELXS97* (Sheldrick, 2008 ▶); program(s) used to refine structure: *SHELXL97* (Sheldrick, 2008 ▶); molecular graphics: *SHELXTL* (Sheldrick, 2008 ▶); software used to prepare material for publication: *SHELXTL*.

## Supplementary Material

Crystal structure: contains datablocks I, global. DOI: 10.1107/S1600536809044444/hb5174sup1.cif

Structure factors: contains datablocks I. DOI: 10.1107/S1600536809044444/hb5174Isup2.hkl

Additional supplementary materials:  crystallographic information; 3D view; checkCIF report

## Figures and Tables

**Table 1:** Hydrogen-bond geometry (Å, °)

*D*—H⋯*A*	*D*—H	H⋯*A*	*D*⋯*A*	*D*—H⋯*A*
N1—H1*A*⋯N3^i^	0.86	2.14	2.999 (3)	177
N1—H1*B*⋯O3^ii^	0.86	1.98	2.830 (3)	173
N2—H2*A*⋯O4^ii^	0.86	1.74	2.593 (3)	175
N4—H4*A*⋯N6^iii^	0.86	2.19	3.046 (3)	175
N4—H4*B*⋯O1^iv^	0.86	2.00	2.851 (3)	173
N5—H5*A*⋯O2^iv^	0.86	1.75	2.606 (3)	174
C1—H1*C*⋯O4^ii^	0.96	2.56	3.355 (4)	140
C7—H7*A*⋯O2^iv^	0.96	2.57	3.355 (4)	139

Symmetry codes: (i) 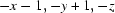; (ii) 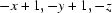; (iii) 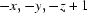; (iv) 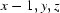.

**Fig. 1.**
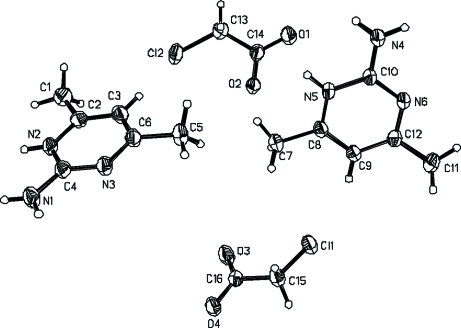
The molecular structure of (I) showing 30% probability displacement ellipsoids.

## supplementary crystallographic information

### Comment

As useful precursors to potentially bioactive pyrimidine derivatives, methylpyrimidine has attracted considerable attention for many years (Xue *et al.*, 1993). In recent years, new complexes of pyrimidine have been synthesized (Hemamalini *et al.*, 2005). The title compound(I), was synthesized and we report herein its crystal structure (Fig. 1).

There are two 2-amino-4,6-dimethylpyrimidine cations and two chloracetate anions in the asymmetric unit. In the crystal structure, cations and anions are linked by intermolecular N—H···O and N—H···N hydrogen bonds to form a two-dimensional network. Additional stabilization is provided by weak intermolecular C—H···O interactions.

### Experimental

A mixture of guanidine hydrochloride (0.1 mol), acetyl acetone (0.2 mol), sodium carbonate (0.03 mol) and 2-chloroacetic acid (0.1 mol) was stirred with water (30 ml) for 3 h to afford the title compound (yield 67%). Colourless blocks of (I) were obtained by recrystallization of the title compound from water at room temperature.

### Refinement

H atoms bonded to C atoms were fixed geometrically and and included in a riding-model approximation with C—H = 0.93–0.96 Å and *U*_iso_(H)=1.2–1.5*U*_eq_(C).

### Crystal data

**Table:**

C_6_H_10_N_3_^+^·C_2_H_2_ClO_2_^−^	*Z* = 4
*M**_r_* = 217.66	*F*(000) = 456
Triclinic, *P*1	*D*_x_ = 1.368 Mg m^−^^3^
Hall symbol: -P 1	Mo *K*α radiation, λ = 0.71073 Å
*a* = 4.4560 (9) Å	Cell parameters from 3452 reflections
*b* = 12.302 (3) Å	θ = 3.2–27.5°
*c* = 19.441 (4) Å	µ = 0.34 mm^−^^1^
α = 92.90 (3)°	*T* = 293 K
β = 96.53 (3)°	Block, colourless
γ = 91.15 (3)°	0.20 × 0.15 × 0.11 mm
*V* = 1057.1 (4) Å^3^	

### Data collection

**Table:**

Bruker SMART CCD diffractometer	3452 reflections with *I* > 2σ(*I*)
Radiation source: fine-focus sealed tube	*R*_int_ = 0.028
graphite	θ_max_ = 27.5°, θ_min_ = 3.2°
ω scans	*h* = −5→5
10303 measured reflections	*k* = −15→15
4761 independent reflections	*l* = −25→25

### Refinement

**Table:**

Refinement on *F*^2^	Primary atom site location: structure-invariant direct methods
Least-squares matrix: full	Secondary atom site location: difference Fourier map
*R*[*F*^2^ > 2σ(*F*^2^)] = 0.055	Hydrogen site location: inferred from neighbouring sites
*wR*(*F*^2^) = 0.195	H-atom parameters constrained
*S* = 1.08	*w* = 1/[σ^2^(*F*_o_^2^) + (0.1056*P*)^2^ + 0.3945*P*] where *P* = (*F*_o_^2^ + 2*F*_c_^2^)/3
4761 reflections	(Δ/σ)_max_ < 0.001
253 parameters	Δρ_max_ = 0.42 e Å^−^^3^
0 restraints	Δρ_min_ = −0.43 e Å^−^^3^

### Special details

**Table:**

Geometry. All e.s.d.'s (except the e.s.d. in the dihedral angle between two l.s. planes) are estimated using the full covariance matrix. The cell e.s.d.'s are taken into account individually in the estimation of e.s.d.'s in distances, angles and torsion angles; correlations between e.s.d.'s in cell parameters are only used when they are defined by crystal symmetry. An approximate (isotropic) treatment of cell e.s.d.'s is used for estimating e.s.d.'s involving l.s. planes.
Refinement. Refinement of *F*^2^ against ALL reflections. The weighted *R*-factor *wR* and goodness of fit *S* are based on *F*^2^, conventional *R*-factors *R* are based on *F*, with *F* set to zero for negative *F*^2^. The threshold expression of *F*^2^ > σ(*F*^2^) is used only for calculating *R*-factors(gt) *etc*. and is not relevant to the choice of reflections for refinement. *R*-factors based on *F*^2^ are statistically about twice as large as those based on *F*, and *R*- factors based on ALL data will be even larger.

### Fractional atomic coordinates and isotropic or equivalent isotropic displacement parameters (Å^2^)

**Table:**

	*x*	*y*	*z*	*U*_iso_*/*U*_eq_	
Cl1	0.92264 (17)	0.01264 (6)	0.17209 (4)	0.0623 (2)	
O4	0.7689 (5)	0.14297 (17)	−0.00572 (10)	0.0681 (6)	
C16	0.8811 (6)	0.13347 (19)	0.05672 (13)	0.0463 (5)	
O3	1.0794 (5)	0.19170 (18)	0.08873 (11)	0.0742 (7)	
C15	0.7353 (7)	0.0407 (2)	0.09016 (14)	0.0616 (7)	
H15A	0.7320	−0.0243	0.0596	0.074*	
H15B	0.5276	0.0584	0.0954	0.074*	
Cl2	0.8148 (2)	0.60659 (7)	0.34169 (5)	0.0862 (3)	
O2	0.9854 (5)	0.38470 (15)	0.37047 (10)	0.0594 (5)	
C14	0.8160 (6)	0.4192 (2)	0.41493 (13)	0.0497 (6)	
O1	0.7445 (6)	0.36870 (18)	0.46320 (12)	0.0798 (7)	
C13	0.6862 (8)	0.5308 (2)	0.40795 (16)	0.0632 (7)	
H13A	0.7342	0.5722	0.4518	0.076*	
H13B	0.4680	0.5230	0.3993	0.076*	
N5	0.1724 (4)	0.18552 (15)	0.37450 (10)	0.0418 (4)	
H5A	0.1093	0.2511	0.3762	0.050*	
N6	0.1954 (5)	0.01175 (16)	0.42234 (10)	0.0467 (5)	
C10	0.1065 (6)	0.11536 (19)	0.42261 (12)	0.0436 (5)	
C9	0.4323 (6)	0.0461 (2)	0.32177 (13)	0.0502 (6)	
H9A	0.5446	0.0210	0.2870	0.060*	
C12	0.3572 (6)	−0.02223 (19)	0.37232 (13)	0.0477 (5)	
C8	0.3363 (5)	0.15178 (19)	0.32440 (12)	0.0430 (5)	
N4	−0.0552 (6)	0.15065 (18)	0.47176 (12)	0.0610 (6)	
H4A	−0.1012	0.1075	0.5026	0.073*	
H4B	−0.1145	0.2167	0.4729	0.073*	
C7	0.4002 (7)	0.2310 (2)	0.27171 (13)	0.0554 (6)	
H7A	0.3149	0.2998	0.2829	0.083*	
H7B	0.3119	0.2038	0.2266	0.083*	
H7C	0.6146	0.2402	0.2718	0.083*	
C11	0.4550 (8)	−0.1383 (2)	0.37303 (18)	0.0689 (8)	
H11A	0.3841	−0.1721	0.4118	0.103*	
H11B	0.6715	−0.1400	0.3770	0.103*	
H11C	0.3719	−0.1768	0.3308	0.103*	
N2	0.0615 (5)	0.69331 (16)	0.07225 (10)	0.0457 (5)	
H2A	0.1088	0.7470	0.0486	0.055*	
N3	−0.2359 (5)	0.53279 (17)	0.07940 (11)	0.0489 (5)	
C6	−0.0956 (6)	0.5215 (2)	0.14272 (13)	0.0493 (6)	
N1	−0.2951 (5)	0.63311 (18)	−0.01745 (11)	0.0566 (6)	
H1A	−0.4349	0.5876	−0.0354	0.068*	
H1B	−0.2464	0.6878	−0.0401	0.068*	
C4	−0.1547 (5)	0.61899 (19)	0.04524 (12)	0.0443 (5)	
C2	0.2013 (5)	0.6821 (2)	0.13654 (13)	0.0472 (5)	
C3	0.1249 (6)	0.5956 (2)	0.17351 (14)	0.0520 (6)	
H3B	0.2183	0.5868	0.2181	0.062*	
C1	0.4302 (6)	0.7682 (2)	0.16441 (15)	0.0594 (7)	
H1C	0.4485	0.8204	0.1300	0.089*	
H1D	0.6220	0.7355	0.1759	0.089*	
H1E	0.3673	0.8039	0.2052	0.089*	
C5	−0.1883 (8)	0.4234 (2)	0.17923 (16)	0.0657 (8)	
H5B	−0.3422	0.3821	0.1499	0.099*	
H5C	−0.2654	0.4466	0.2216	0.099*	
H5D	−0.0162	0.3788	0.1896	0.099*	

### Atomic displacement parameters (Å^2^)

**Table:**

	*U*^11^	*U*^22^	*U*^33^	*U*^12^	*U*^13^	*U*^23^
Cl1	0.0689 (4)	0.0673 (4)	0.0516 (4)	−0.0052 (3)	0.0041 (3)	0.0202 (3)
O4	0.0883 (15)	0.0633 (12)	0.0497 (11)	−0.0230 (11)	−0.0076 (10)	0.0170 (9)
C16	0.0500 (13)	0.0415 (12)	0.0478 (13)	−0.0017 (10)	0.0065 (10)	0.0057 (10)
O3	0.0887 (15)	0.0727 (14)	0.0573 (12)	−0.0334 (12)	−0.0100 (11)	0.0213 (10)
C15	0.0666 (17)	0.0649 (17)	0.0515 (15)	−0.0184 (14)	−0.0035 (12)	0.0182 (13)
Cl2	0.1195 (7)	0.0527 (4)	0.0972 (7)	0.0286 (4)	0.0432 (5)	0.0302 (4)
O2	0.0773 (13)	0.0450 (10)	0.0609 (11)	0.0132 (9)	0.0229 (10)	0.0130 (8)
C14	0.0569 (14)	0.0407 (12)	0.0512 (14)	0.0017 (11)	0.0031 (11)	0.0057 (10)
O1	0.1139 (19)	0.0612 (13)	0.0750 (14)	0.0228 (12)	0.0437 (13)	0.0261 (11)
C13	0.0779 (19)	0.0520 (15)	0.0638 (17)	0.0163 (14)	0.0193 (14)	0.0105 (13)
N5	0.0532 (11)	0.0323 (9)	0.0405 (10)	0.0009 (8)	0.0054 (8)	0.0097 (7)
N6	0.0617 (12)	0.0356 (10)	0.0449 (10)	0.0052 (9)	0.0101 (9)	0.0118 (8)
C10	0.0554 (13)	0.0375 (11)	0.0387 (11)	0.0014 (10)	0.0054 (9)	0.0096 (9)
C9	0.0628 (15)	0.0461 (13)	0.0436 (12)	0.0069 (11)	0.0116 (11)	0.0071 (10)
C12	0.0581 (14)	0.0393 (12)	0.0463 (13)	0.0068 (10)	0.0044 (10)	0.0076 (9)
C8	0.0476 (12)	0.0429 (12)	0.0383 (11)	−0.0006 (9)	0.0008 (9)	0.0096 (9)
N4	0.0932 (17)	0.0420 (11)	0.0545 (13)	0.0128 (11)	0.0300 (12)	0.0158 (9)
C7	0.0702 (16)	0.0512 (14)	0.0475 (13)	−0.0013 (12)	0.0140 (12)	0.0159 (11)
C11	0.094 (2)	0.0426 (14)	0.074 (2)	0.0197 (14)	0.0196 (17)	0.0118 (13)
N2	0.0515 (11)	0.0420 (10)	0.0458 (11)	−0.0001 (9)	0.0120 (8)	0.0094 (8)
N3	0.0563 (12)	0.0423 (10)	0.0505 (12)	0.0016 (9)	0.0129 (9)	0.0112 (9)
C6	0.0574 (14)	0.0451 (12)	0.0490 (13)	0.0119 (11)	0.0147 (11)	0.0138 (10)
N1	0.0679 (14)	0.0504 (12)	0.0507 (12)	−0.0145 (11)	0.0014 (10)	0.0147 (9)
C4	0.0480 (12)	0.0426 (12)	0.0443 (12)	0.0013 (10)	0.0116 (10)	0.0070 (9)
C2	0.0444 (12)	0.0497 (13)	0.0494 (13)	0.0098 (10)	0.0111 (10)	0.0051 (10)
C3	0.0547 (14)	0.0561 (14)	0.0472 (13)	0.0110 (12)	0.0089 (11)	0.0118 (11)
C1	0.0571 (15)	0.0599 (16)	0.0599 (16)	0.0006 (13)	0.0025 (12)	0.0015 (13)
C5	0.085 (2)	0.0530 (15)	0.0628 (17)	0.0037 (14)	0.0126 (15)	0.0231 (13)

### Geometric parameters (Å, °)

**Table:**

Cl1—C15	1.766 (3)	C7—H7A	0.9600
O4—C16	1.271 (3)	C7—H7B	0.9600
C16—O3	1.219 (3)	C7—H7C	0.9600
C16—C15	1.512 (3)	C11—H11A	0.9600
C15—H15A	0.9700	C11—H11B	0.9600
C15—H15B	0.9700	C11—H11C	0.9600
Cl2—C13	1.767 (3)	N2—C2	1.347 (3)
O2—C14	1.273 (3)	N2—C4	1.357 (3)
C14—O1	1.220 (3)	N2—H2A	0.8600
C14—C13	1.507 (4)	N3—C6	1.331 (3)
C13—H13A	0.9700	N3—C4	1.344 (3)
C13—H13B	0.9700	C6—C3	1.389 (4)
N5—C8	1.337 (3)	C6—C5	1.505 (3)
N5—C10	1.356 (3)	N1—C4	1.326 (3)
N5—H5A	0.8600	N1—H1A	0.8600
N6—C12	1.331 (3)	N1—H1B	0.8600
N6—C10	1.342 (3)	C2—C3	1.372 (4)
C10—N4	1.322 (3)	C2—C1	1.493 (4)
C9—C8	1.378 (4)	C3—H3B	0.9300
C9—C12	1.389 (3)	C1—H1C	0.9600
C9—H9A	0.9300	C1—H1D	0.9600
C12—C11	1.501 (4)	C1—H1E	0.9600
C8—C7	1.495 (3)	C5—H5B	0.9600
N4—H4A	0.8600	C5—H5C	0.9600
N4—H4B	0.8600	C5—H5D	0.9600
			
O3—C16—O4	126.0 (2)	C8—C7—H7C	109.5
O3—C16—C15	121.5 (2)	H7A—C7—H7C	109.5
O4—C16—C15	112.5 (2)	H7B—C7—H7C	109.5
C16—C15—Cl1	113.42 (19)	C12—C11—H11A	109.5
C16—C15—H15A	108.9	C12—C11—H11B	109.5
Cl1—C15—H15A	108.9	H11A—C11—H11B	109.5
C16—C15—H15B	108.9	C12—C11—H11C	109.5
Cl1—C15—H15B	108.9	H11A—C11—H11C	109.5
H15A—C15—H15B	107.7	H11B—C11—H11C	109.5
O1—C14—O2	125.4 (2)	C2—N2—C4	119.3 (2)
O1—C14—C13	116.1 (2)	C2—N2—H2A	120.4
O2—C14—C13	118.5 (2)	C4—N2—H2A	120.4
C14—C13—Cl2	115.3 (2)	C6—N3—C4	117.5 (2)
C14—C13—H13A	108.5	N3—C6—C3	122.1 (2)
Cl2—C13—H13A	108.5	N3—C6—C5	116.2 (2)
C14—C13—H13B	108.5	C3—C6—C5	121.7 (2)
Cl2—C13—H13B	108.5	C4—N1—H1A	120.0
H13A—C13—H13B	107.5	C4—N1—H1B	120.0
C8—N5—C10	119.4 (2)	H1A—N1—H1B	120.0
C8—N5—H5A	120.3	N1—C4—N3	118.6 (2)
C10—N5—H5A	120.3	N1—C4—N2	118.3 (2)
C12—N6—C10	117.6 (2)	N3—C4—N2	123.1 (2)
N4—C10—N6	118.5 (2)	N2—C2—C3	119.7 (2)
N4—C10—N5	118.3 (2)	N2—C2—C1	116.9 (2)
N6—C10—N5	123.2 (2)	C3—C2—C1	123.4 (2)
C8—C9—C12	118.4 (2)	C2—C3—C6	118.4 (2)
C8—C9—H9A	120.8	C2—C3—H3B	120.8
C12—C9—H9A	120.8	C6—C3—H3B	120.8
N6—C12—C9	121.8 (2)	C2—C1—H1C	109.5
N6—C12—C11	116.7 (2)	C2—C1—H1D	109.5
C9—C12—C11	121.5 (2)	H1C—C1—H1D	109.5
N5—C8—C9	119.7 (2)	C2—C1—H1E	109.5
N5—C8—C7	117.8 (2)	H1C—C1—H1E	109.5
C9—C8—C7	122.5 (2)	H1D—C1—H1E	109.5
C10—N4—H4A	120.0	C6—C5—H5B	109.5
C10—N4—H4B	120.0	C6—C5—H5C	109.5
H4A—N4—H4B	120.0	H5B—C5—H5C	109.5
C8—C7—H7A	109.5	C6—C5—H5D	109.5
C8—C7—H7B	109.5	H5B—C5—H5D	109.5
H7A—C7—H7B	109.5	H5C—C5—H5D	109.5

### Hydrogen-bond geometry (Å, °)

**Table:**

*D*—H···*A*	*D*—H	H···*A*	*D*···*A*	*D*—H···*A*
N1—H1A···N3^i^	0.86	2.14	2.999 (3)	177
N1—H1B···O3^ii^	0.86	1.98	2.830 (3)	173
N2—H2A···O4^ii^	0.86	1.74	2.593 (3)	175
N4—H4A···N6^iii^	0.86	2.19	3.046 (3)	175
N4—H4B···O1^iv^	0.86	2.00	2.851 (3)	173
N5—H5A···O2^iv^	0.86	1.75	2.606 (3)	174
C1—H1C···O4^ii^	0.96	2.56	3.355 (4)	140
C7—H7A···O2^iv^	0.96	2.57	3.355 (4)	139

Symmetry codes: (i) −*x*−1, −*y*+1, −*z*; (ii) −*x*+1, −*y*+1, −*z*; (iii) −*x*, −*y*, −*z*+1; (iv) *x*−1, *y*, *z*.
